# Optimizing the Texturing Parameters of Concrete Pavement by Balancing Skid-Resistance Performance and Driving Stability

**DOI:** 10.3390/ma14206137

**Published:** 2021-10-15

**Authors:** Jiangmiao Yu, Binhui Zhang, Peiqi Long, Bo Chen, Feng Guo

**Affiliations:** 1 School of Civil Engineering and Transportation, South China University of Technology, Guangzhou 510006, China; yujm@scut.edu.cn (J.Y.); ctbhzhang@scut.edu.cn (B.Z.); 2 Foshan Branch, Suzhou Planning & Design Research Institute Co., Ltd., Foshan 528300, China; longpeiqi163@163.com; 3 Department of Civil and Environmental Engineering, University of South Carolina, Columbia, SC 29201, USA; fengg@email.sc.edu

**Keywords:** skid-resistance performance, orthogonal test, abrasion test, groove parameters

## Abstract

Curved texturing is an effective technique to improve the skid-resistance performance of concrete pavements, which relies on the suitable combination of the groove parameters. This study aims to optimize these parameters with the consideration of skid-resistance performance and driving stability. A pressure film was adopted to obtain the contact stress distribution at the tire–pavement interface. The evaluated indicator of the stress concentration coefficient was established, and the calculation method for the stationary steering resistance torque was optimized based on actual tire–pavement contact characteristics. Test samples with various groove parameters were prepared use self-design molds to evaluate the influence degree of each groove parameter at different levels on the skid-resistance performance through orthogonal and abrasion resistance tests. The results showed that the groove depth and groove spacing had the most significant influence on the stress concentration coefficient and stationary steering resistance torque, respectively, with the groove depth having the most significant influence on the texture depth. Moreover, the driving stability and durability of the skid-resistance performance could be balanced by optimizing the width of the groove group. After analyzing and comprehensively comparing the influences of various parameters, it was found the parameter combination with width, depth, spacing, and the groove group width, respectively, in 8 mm, 3 mm, 15 mm, and 50 mm can balance the skid-resistance performance and driving stability. The actual engineering results showed that the R^2^ of the fitting between the stress concentration coefficient and SFC (measured at 60 km/h) was 0.871, which proved the effectiveness of the evaluation index proposed in this paper.

## 1. Introduction

The anti-skid performance of concrete pavements has been the focus of research. Moreover, a good skid-resistance performance can help to effectively reduce the slip accident rate. Conventional techniques of improving the anti-skid performance include dragging, grinding, and grooving [1,2,3,4,5,6]. Grooving is the most commonly used in engineering construction, and the design of the groove dimension and shape is key [7,8,9,10]. Fwa and Ong established a simulation model and concluded that the skid-resistance effect is significant when using a rectangular groove with width, depth, and spacing in the ranges of 2–10, 1–10, and 5–25 mm, respectively [11]. By adopting a continuous friction tester to measure the sideway-force coefficient (SFC) on various pavement surfaces at the same vehicle speed, Zhang recommended a rectangular groove with a large center spacing of 25 cm [12]. With the use of diamond saw blades to cut rectangular grooves on cured concrete pavements, the grooving technology can help improve the macrotexture of the pavement; however, the cutting surface is relatively flat, which reduces the effective tire–pavement contact area and has no significant effect on improving the microtexture [13,14,15,16,17]. Related research combined diamond grinding and grooving technology to improve the microtexture and macrotexture of the surface; however, this method has many drawbacks such as poor maneuverability and high costs [18,19]. In 2012, a new texturing technology was introduced in China to improve the skid-resistance performance of concrete pavements by carving dense longitudinal corrugated grooves on a cured pavement. Additionally, the average SFC of the curved textured pavement reached 68, which is higher than that of rectangular grooved pavements (approximately in the range of 15–30%). A fuller contact provides a greater lateral force, resulting in a more pronounced wheel shimmy [20,21]. A survey on a completed textured pavement found that some vehicles have poor driving stability at speeds close to 100 km/h. The shimmy is associated with complex nonlinear dynamics of the influencing factors, and the mechanical parameters are difficult to measure when a vehicle is in motion [22,23,24]. Related studies have shown that the tire–pavement friction is a factor influencing wheel shimmy [25,26,27].

The skid resistance force has been considered a critical indicator of the concrete pavement performance by the Portland Cement Association (PCA) and American Association of State Highway and Transportation Officials (AASHTO) [28,29,30,31,32]. Related studies have proven that the skid-resistance is influenced by many factors, including the pavement surface texture, tire type, tire–pavement friction, or other factors such as the speed, water film depth, and temperature, among which the tire–pavement friction is a primary factor [33,34,35,36]. Skid resistance is generally evaluated directly by the friction coefficient. The conventional measurement methods of the pavement skid resistance include the sand patch test, outflow meter, British Pendulum tester (BPT) and dynamic friction tester (DFT) testing, which mainly focus on the characterization of pavement macrotexture by using the volumetric index under specific conditions (certain load, test speed, friction mode, water-film thickness) [37,38,39]. Some novel measurement methods including but not limited to laser scanning, mechanical stylus, and image processing have also been developed to describe the surface texture characterization more accurately by capturing and showing the 2-D curve or 3-D surface texture and morphology within the measured region [4,40]. Many experimental results have shown that the groove parameters significantly influence the tire–pavement contact surface and that a suitable combination of parameters can help effectively improve the skid-resistance performance [41,42,43]. Although the relationship between the contact stress distribution and the skid-resistance performance has been studied, the actual characteristics at the tire–pavement contact have been largely simplified. The actual tire–pavement contact stress cannot be obtained using the conventional evaluation method of the skid resistance because of technical limitations.

Additionally, there is currently no clear standard for texturing technology. The selection of the groove parameters of the textured pavement is mainly based on engineering experience, and the lack of long-term monitoring data of related projects makes it difficult to verify the reliability of the empirical parameters. Therefore, it is necessary to study the contact stress distribution characteristics and develop a more accurate measurement method to evaluate the skid-resistance performance. 

Based on tire–pavement friction, the stress concentration indicators were proposed, and the calculation method for the stationary steering resistance torque was optimized. In addition, an L9 (3^4^) orthogonal table was determined on the basis of the selected parameters and levels, and a range analysis was conducted using these evaluation indices. Next, the effect of the groove group width (GGW) was studied, and an optimal combination of the parameters was determined through a comprehensive analysis of orthogonal and abrasion resistance test results. Finally, the skid-resistance performance under a set of optimized parameters was verified by actual project cases.

## 2. Objectives and Scope of this Study 

The objectives and scope of this study are as follows:

(1) An evaluation method for the contact mechanics was established to describe the skid resistance and influence of the cement pavement on the driving stability, which should be verified based on engineering test results.
(2) A set of small texturing equipment that can help prepare specimens with different texture parameters in the laboratory was developed. Pressure-sensitive films were used to obtain the contact stress distribution between the tire and different pavements. Texture parameters that can provide a balance between driving stability and skid resistance performance are recommended by conducting orthogonal and kneading tests.

## 3. Methodology

### 3.1. Tire–Pavement Friction

**Figure 1:**
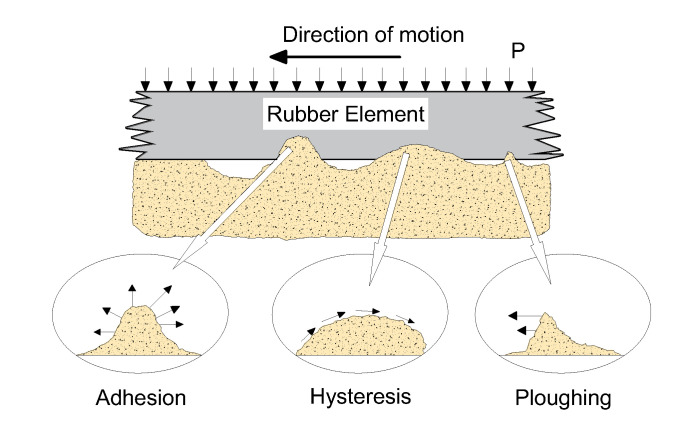
Tire–pavement friction.

Persson concluded that the tire–pavement contact is incomplete [44]. The embedding of the convex texture makes the tire deform, resulting in a stress concentration at the contact interface. Moore explained the friction phenomenon between tire and pavement surface. The adhesion (*F_a_*), hysteresis (*F_h_*), and ploughing (*F_p_*) components of the frictional forces in elastomers are shown in Figure 1 and Equation (1).

(1) $$F=F_{a}+F_{h}+F_{p}$$

An adhesive friction is mainly produced between the rubber and microtexture, which comprises 90% of the friction at low speeds [45,46]. The stress on the asperities is proportional to the adhesion friction, and a greater adhesion provides a better skid resistance performance. The adhesion friction function is expressed in Equation (2).

(2) $$F_{a}=K_{1}K_{2}\sigma_{m}\frac{N}{H}\tan\delta$$

where *K*_1_ and *K*_2_ are constants, *σ_m_* is the maximum normal stress at the top of the asperity, *N* is the normal load, *H* is the rubber hardness, and *tanδ* is the tangent modulus of rubber.

When vehicles pass across the asperities of a rough surface pavement, the hysteresis component reflects the energy lost during this process, as the rubber is alternately compressed and decompressed [47,48]. The hysteresis friction function is expressed in Equation (3).

(3) $$F_{h}=c\sum_{i=1}^{n}\left(E_{ci}-E_{ei}\right)$$

where *c* is a constant, *E_ci_* − *E_ei_* is the energy loss of the tire rubber on a single asperity surface.

The asperities on the pavement surface will have a microcutting effect on the tire, and the ploughing force is shown in Equation (4).

(4) $$F_{p}=K_{3}htg(\delta )T_{\max}=K_{3}K_{4}{\left|\frac{P}{tg\frac{\theta }{2}}\right|}^{\frac{1}{2}}tg(\delta )T_{\max}$$

where *K*_3_ and *K*_4_ are constants, *N* is the normal stress acting on the asperity; *θ* is the apex angle of the simplified asperity profile; *h* is the depth to which the asperity penetrates the rubber; *tgδ* is the rubber tangent modulus; *T_max_* is the maximum tangential stress that breaks the rubber molecular chain.

### 3.2. Pressure Film Testing

**Figure 2:**
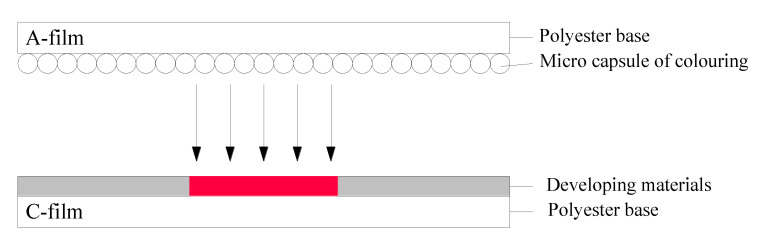
Working principle of a pressure film.

The pressure film (Figure 2) can accurately measure the contact area and the pressure distribution (the minimum effective measurement is 0.125 mm^2^). In this study, double-slice pressure films were chosen, including an A-film with a color generation agent and a C-film with a color developer. Under the application of pressure, the pressure level was described in terms of the color density. Due to the limited range, a complete tire–pavement contact stress cannot be obtained by adopting a single-range pressure film. Therefore, various specification films were adopted, including LLLW (Ultra Super Low Pressure) (0.2–0.6 MPa), LLW (Super Low Pressure) (0.5–2.5 MPa), and LW (Low Pressure) (2.5–10 MPa).

The stress distribution information of the contact interface was stored in a 2D matrix after processing, as shown in Equation (5).

(5) $$F\left(X,Y\right)=\left[\begin{array}{cccc} f\left(0,0\right) & f\left(0,1\right) & \ldots & f\left(0,n\right)\\ f\left(1,0\right) & \ldots & \ldots & \ldots \\ \ldots & \ldots & \ldots & \ldots \\ f\left(m,0\right) & \ldots & \ldots & f\left(m,n\right) \end{array}\right]$$

where *F (X, Y)* is the overall normal stress acting on the contact area, and *f* (*m*, *n*) is the mean contact stress at the measurement point.

**Figure 3:**
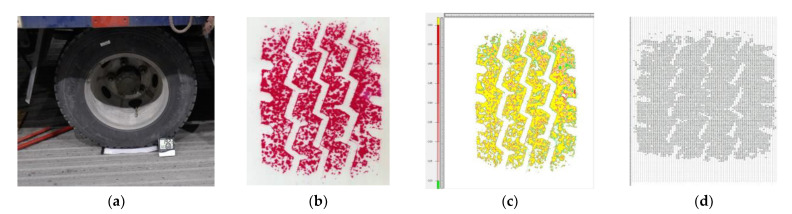
Schematics of pressure film operation process: (**a**) loading; (**b**) tire mark; (**c**) scanning image; (**d**) numerical statistics of contact stress.

The use of the pressure film is as follows:

(1) The film is placed between the tire and the road and statically loaded for more than two mins (Figure 3a).
(2) The temperature and humidity of the test site are recorded, and the correct model of the pressure and color density is determined.
(3) After the color reaction is complete, the test film is calibrated and scanned and identified in the FPD-8010E (Version 1.1, 2007, FIJIFILM Corporation; Tokyo, Japan) dedicated software (Figure 3b,c).
(4) The test results corresponding to the different specifications of the pressure film are analyzed in MATLAB(Version 9.1, 2016, MathWorks company; Natick, MA, USA) and a numerical quantification and statistical analysis is performed (Figure 3d).

### 3.3. Stress Concentration Effect

**Figure 4:**
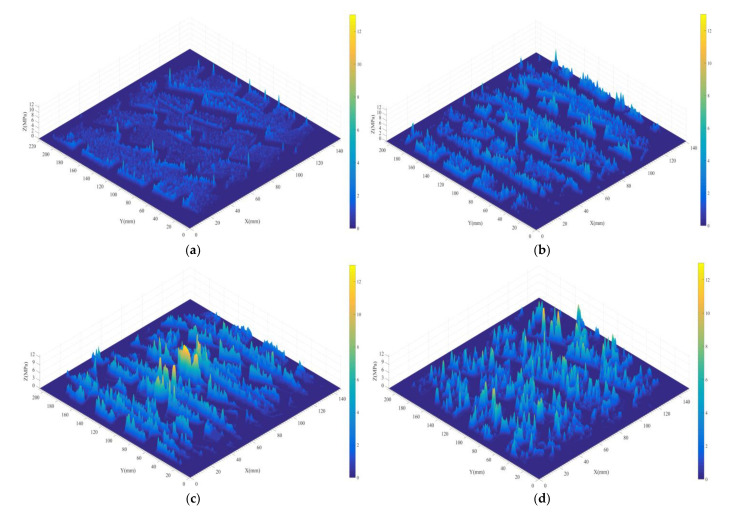
Tire contact stress of different pavements: (**a**) concrete pavements with no grooves, (**b**) rectangular grooves, (**c**) curved grooves, (**d**) asphalt pavement without grooves.

**Figure 5:**
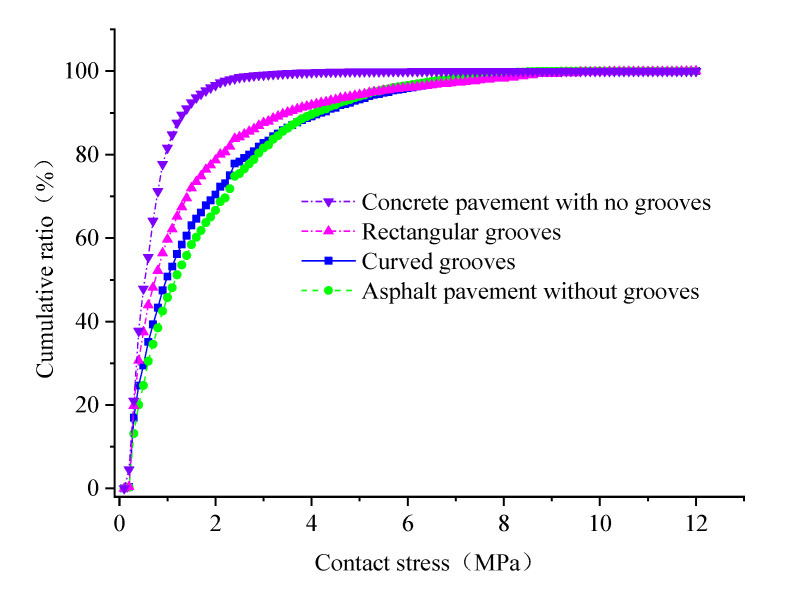
Contact stress distribution on different pavements.

To visually show the difference in the stress distribution, the contact pressure distributions of a pavement with no grooves, a rectangular groove pavement (groove width: 4 mm, groove depth: 4 mm, spacing: 25 mm), a curved groove pavement (groove width: 8 mm, groove depth: 1 mm, spacing: 8 mm), and an asphalt pavement (AC-16) were obtained, as shown at Figure 4, and the contact stress was divided into several parts in steps of 0.1 MPa. The proportion of the stress distribution area of each part in the total effective contact area is counted, and the cumulative proportion is shown in Figure 5. The contact stress of concrete pavements with no grooves, rectangular grooves, curved grooves, and asphalt pavement without grooves are mainly concentrated in 0~3 MPa, 0~7 Mpa, 0~8 MPa, and 0~10 MPa, respectively. According to the results of Figure 5, the smooth concrete slab is flatter and more fully in contact with vehicle tire, resulting in a more even contact stress distribution of tire; the values of contact stress are mainly less than 1.8 MPa. The contact between the AC-16 asphalt pavement and vehicle tire produced a larger stress concentration phenomenon, with the values of contact stress mainly more than 6 MPa; this phenomenon is consistent with the findings of the literature [40]. As the roughness of the pavement construction increases, the embedded effect on the tire becomes more significant, while the top of the construction pierces the tread rubber to the greatest depth, thus generating a significant stress concentration phenomenon. It is clear that the degree of dispersion of the stress distribution is related to the surface texture characteristics.

The Weibull function (Equation (6)) is widely used in material science to characterize the uniformity of the material strength distribution. *c* is called the Weibull modulus. The higher the *c* value, the lower the dispersion degree of the material and the better the uniformity.

(6) $$F(x)=\left\{\begin{array}{cc} 1-\exp\left.\left\{-{\left(\frac{x-a}{b}\right)}^{c}\right.\right\}, & x\geq a\\ 0, & x<a \end{array}\right.\left(b,c>0\right)$$

where *a* is the position parameter, *b* is the scale parameter, and *c* is the shape parameter of the Weibull distribution (also known as the Weibull modulus).

Based on the significance of the Weibull modulus in material science, the value of the Weibull modulus was adopted to characterize the degree of dispersion of the contact stress. Previous studies have shown that the coarse particles and texture depth of a pavement structure directly affect the degree of stress dispersion [21]. Table 1 shows that the smooth concrete pavement has the highest Weibull modulus value, followed by the rectangular grooved pavement, curved textured pavement, and asphalt pavement, in that order. In addition, the non-uniformity of the textured pavement structure is better than that of the grooved pavement, indicating that the macro- and micro-textures of the textured pavement are more complex and that the stress distribution is more discrete.

The stress concentration phenomenon is due to tire–pavement friction. Although the Weibull modulus can be used to explain the dispersion of the stress distribution on the contact surface, it cannot describe the degree of stress concentration effect.

To quantify the degree of stress concentration, a stress concentration coefficient was adopted, which is expressed in Equation (7).

(7) $$K_{s}=\frac{\iint {}_{Aʹ}f\left(x,y\right)dxdy}{\iint {}_{A}f\left(x,y\right)dxdy}\times 100\%$$

where *K_S_* is the stress distribution concentration (%), and *A’* is a high-stress area (mm^2^). According to a previous study [46], the stress above 1.8 MPa is defined as high stress. *A* is the actual contact area between the tire and the pavement (mm^2^), and *f (x, y)* is the single-point contact stress measured by the pressure film. 

### 3.4. Stationary Steering Resistance Torque

**Figure 6:**
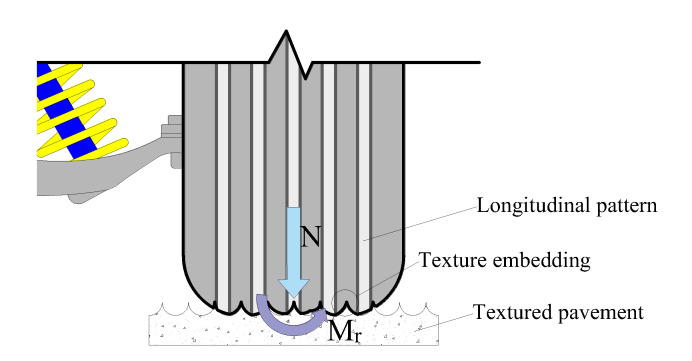
The meshing effect: N is vertical load; Mr is stationary steering resistance torque.

The steering resistance torque of a vehicle is maximum under a static condition, which is affected by the tire–pavement friction and vehicle steering system. The friction coefficient of the steering system is typically considered a constant; therefore, the contact friction is the most important factor [49,50]. Additionally, the mechanical parameters are difficult to measure while the vehicle is in motion; nevertheless, static indicators can indirectly reflect and help evaluate the dynamic process. The tire ground pressure is typically considered a uniform or linear parabolic load distribution, which simplifies the actual contact pressure. This study used the typical distributed stress as the actual contact stress measured by the pressure film. The shape of curved groove is similar to the longitudinal pattern of tire; therefore, the meshing effect between tire and pavement is more significant (Figure 6).

The stationary steering resistance torque was calculated by the effective contact areas and the actual contact stress distribution. The calculation of the microelement of the stationary steering resistance torque at the center of the contact interface is expressed in Equation (8).

(8) $$dM_{r}=r\times dF_{r}=\sqrt{x^{2}+y^{2}}\times \mu \times f(x,y)dxdy$$

Using the MATLAB calculation program to cyclically accumulate the friction torque of each contact unit, we finally obtain the total steering torque value *M_r_*, as expressed in Equation (9).

(9) $$M_{r}=\int dM_{r}=\iint \sqrt{x^{2}+y^{2}}\times \mu \times f(x,y)dxdy$$

where *x* and *y* are the coordinate values of the single-point contact stress (the origin of the coordinate is the contact center point), and the road friction coefficient *μ* was measured using pendulum slip resistance testing equipment; *f* (*x*, *y*) is the single-point contact stress measured by the pressure film.

**Figure 7:**
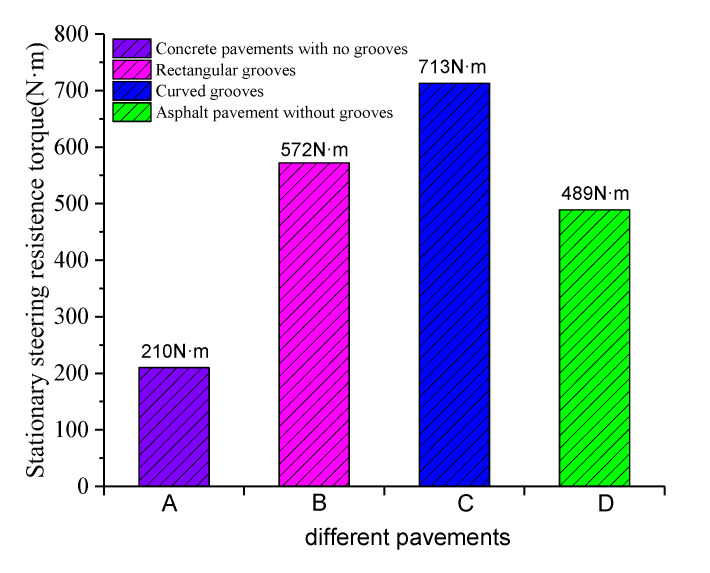
Comparison of stationary steering resistance torque.

By calculating the stationary steering resistance torque on the above four types of pavements, it can be seen from Figure 7 that the stationary steering resistance torque of the textured pavement is greater than that of other types of pavements, which indicates that greater opposite rotary moment of steering wheel is needed to ensure driving stability. Therefore, it is necessary to enhance the driving stability by optimizing texture parameters. 

## 4. Materials and Methods

### 4.1. Materials and Mixture Design

Type P.O42.5 cement (Yunfu, China), medium sand (a fineness modulus of 2.77 and a mud content of 0.8%), limestone aggregate (size ranges of 10–30, 10–20, and 5–10 mm, respectively), and a high-range water reducer (CNF-13, Yunfu, China) were used to prepare the mix. Table 2 and Table 3 list the parameters of the cement and proportion of the mix, respectively.

### 4.2. Sample Preparation

**Figure 8:**
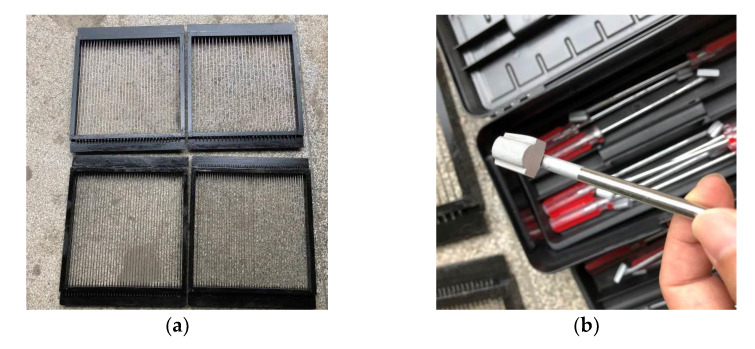
Tester for preparing samples: (**a**) rail mold; (**b**) texturing tool.

Through the calculation of the different curved groove width and depth, a sample preparation tester was developed (Figure 8).

(1) Based on the concrete mix proportion, listed in Table 3, and the groove parameters, listed in Table 4, the samples were molded into a mold of dimensions 300 mm × 300 mm × 50 mm.
(2) Before the concrete sets and solidifies, the slurry on the specimen surface is scraped off.
(3) The rail mold is set, and the steel wire is made to adhere to the sample surface.
(4) A texturing tool is used to carve curved grooves between the two steel wires.
(5) The samples are placed in a standard curing room for seven days, and the texturing tool is used to scrape off the slurry on the groove surface to restore the rough texture.

## 5. Orthogonal Designs

### 5.1. Orthogonal Design

Orthogonal experiments are one of the most commonly used experimental design methods, wherein an orthogonal table is used to scientifically analyze the influence laws of multiple factors. Therefore, the orthogonal test method was chosen in this study to analyze the skid-resistance and driving stability performance of a curved textured pavement with different parameters.

**Figure 9:**
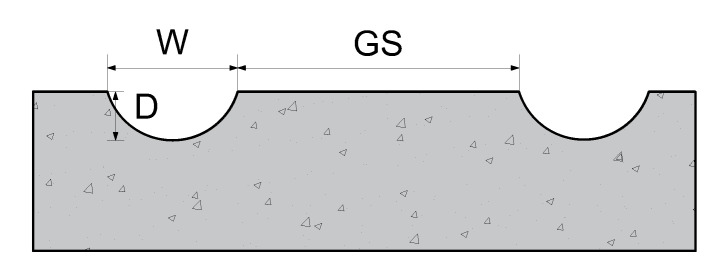
Curved groove parameters (W: the curved groove width, mm; D: the curved groove depth, mm; GS: the curved groove spacing, mm).

Among the impact factors, the curved groove width *W*, depth *D*, and spacing GS were selected; these have a direct impact on the tire–pavement contact interface. Figure 9 shows the diagram of the groove parameters. The three levels of the groove width *W* are 6, 8, and 10 mm, the three levels of the groove depth *D* are 1, 2, and 3 mm, and the three levels of the groove spacing GS are 0, 15, and 25 mm. An L9 (3^4^) orthogonal table, including three factors and three levels, is shown in Table 4.

### 5.2. Analysis of Orthogonal Test of Results

A single wheel load of 15.8 kN and a pressure of 770 kPa were selected for the test, samples with different groove parameters were statically loaded, and then pressure films were adopted to obtain the stress distribution information. The sand paving method and pendulum tester were used to obtain the texture depth TD and lateral friction coefficient, respectively. The stress concentration coefficient *Ks* and the stationary steering resistance torque *M_r_* of the samples were calculated using the method introduced above. Table 5 lists the calculation results.

To evaluate the influence degree of the three factors on the test results, a range analysis of the orthogonal method is necessary. In the calculation of the range analysis, the value *K_ij_* and the influence degree *R_j_* are shown in Equations (10) and (11), respectively.

(10) $$K_{ij}=\sum_{i=1}^{n}y_{ij}$$

(11) $$Rj=\max\{K_{1j},K_{2j},K_{3j}\}-\min\{K_{1j},K_{2j},K_{3j}\}$$

where *i* is the level number; *j* represents the impact factor; *n* = 3; *y* is the test result.

In the range analysis, the value *K_ij_* when *i* = 1, 2, and 3 describes the effect of the factor *j* on the test result. Moreover, the influence degree *R_j_* measures the degree of impact of the factor *j*. Using the analysis method above, the range analysis results with different *K_ij_* and *R_j_* values are shown in Table 6.

As shown in Table 6, the range analysis is implemented at different factors and levels to facilitate a comparative analysis. In terms of the texture depth TD, stress concentration coefficient, and stationary steering resistance torque, the results show that R_D_ > R_GS_ > R_W_, R_D_ > R_W_ > R_GS_, and R_GS_ > R_D_ > R_W_, respectively. 

**Figure 10:**
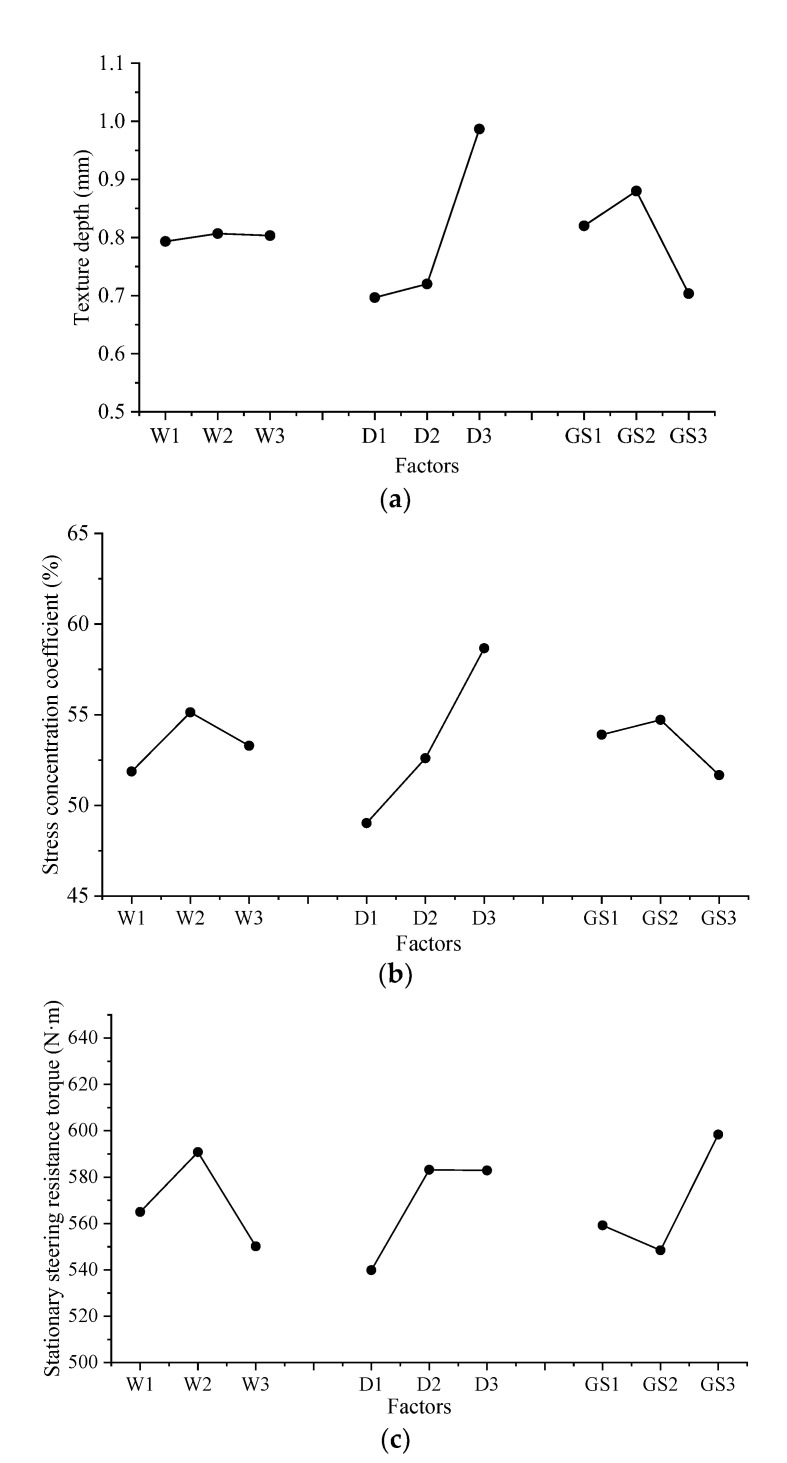
Average value per level for each factor: (**a**) texture depth; (**b**) stress concentration coefficient; (**c**) stationary steering resistance torque.

Based on the selection criteria of the orthogonal test, the factors with the highest influence degree should be selected with an appropriate level, and the less important factors can be arbitrarily selected. Figure 10 shows the average value of each factor at each level for different test results. Figure 10 shows that *Ks* and *Mr* increase first and then decrease with the increase in the groove width, and *TD* and *Ks* increase with the increase in the groove depth. 

For *Ks* and *TD*, to obtain a high value, the level of the groove depth *D* should be D_3_ (3 mm). Considering that the influence of groove spacing on *Mr* is the highest, the level of groove spacing should be GS_2_ (15 mm). At this level, *Mr* takes a small value, which is conducive to the driving stability. Additionally, from Table 6, we find that the groove width is the second most important factor influencing *K_S_*, which is maximum when *W* is W2 (8 mm).

To determine whether the changes in the various factors and test errors significantly affect the test indicators, the analysis of variance was applied to further analyze the test data. The F distribution table was adopted to quantitatively evaluate the significance level of each factor and test the significance of each factor. The commonly used significance levels (α) are 0.01, 0.05, and 0.10, and the critical values are F_0.01_ (2,2) = 99, F_0.05_ (2,2) = 19, and F_0.10_ (2,2) = 9, respectively. F > F_0.01_ (2,2) indicates high significance, F_0.01_ (2,2) > F > F_0.05_ (2,2) indicates moderate significance, and F < F_0.05_ (2,2) indicates no significance. Based on the results of the orthogonal experiment, the SPSS statistical analysis software was used to analyze the data.

Based on the results of the variance analysis listed in Table 7, the three groove parameters are found to have a significant impact on the stress concentration coefficient and stationary steering resistance torque. The groove depth and groove spacing significantly affect the texture depth, whereas the groove width has the least significant impact. Therefore, considering the above analysis, the levels (values) of each factor should be selected as W_2_ (8 mm), D_3_ (3 mm), and GS_2_ (15 mm). The test under these levels can be considered an optimized test. 

To further improve the skid-resistance performance of cement pavements while maintaining the driving stability, the width of the groove group was selected, and the long-term influence corresponding to different texture parameter combinations on the stress concentration coefficient and stationary steering resistance torque is further studied through a kneading test.

## 6. Durability Research Based on Abrasion Test

### 6.1. Design of Abrasion Test

The combination of the texture parameters obtained from the orthogonal test can only reflect the initial skid-resistance performance. The samples (8 mm in width, 3 mm in depth, and 1 mm in spacing) exhibiting the best skid resistance in the forementioned tests were selected, and the groove parameters were further explored by refining the GGW to identify the differences in their skid-resistance durability.

**Figure 11:**
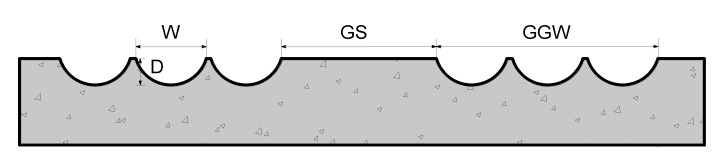
New curved groove parameters (W: the curved groove width, mm; D: the curved groove depth, mm; GS: the curved groove spacing, mm; GGW- the curved groove group width, mm).

**Figure 12:**
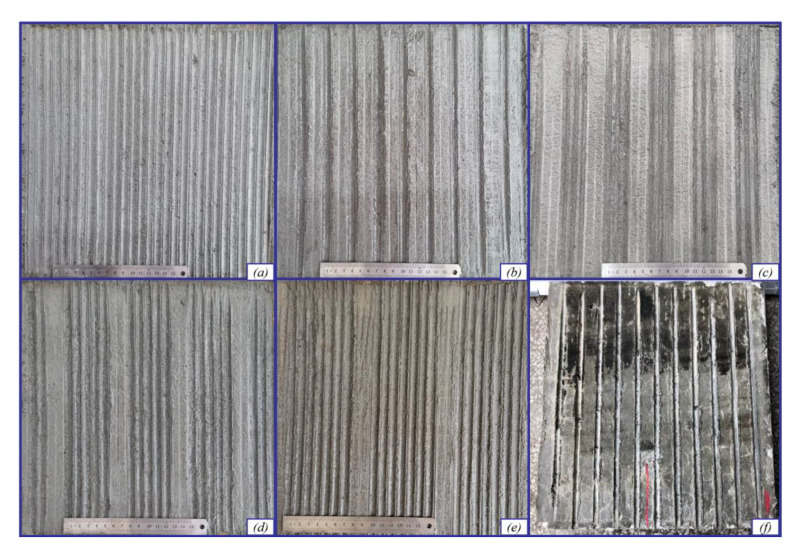
Test specimens and groove size schematic diagram: (**a**) T-1:8-3-0-8; (**b**) T-2:8-3-15-8; (**c**) T-3:8-3-15-30; (**d**) T-4:8-3-15-50; (**e**) T-5:8-3-15-70; (**f**) rectangular groove.

Figure 11 shows the GGW. In accordance with the sample preparation method presented in Section 3.2, six types of cement board samples with different texture parameters were prepared for the experiment, as shown in Figure 12 and Table 8.

**Figure 13:**
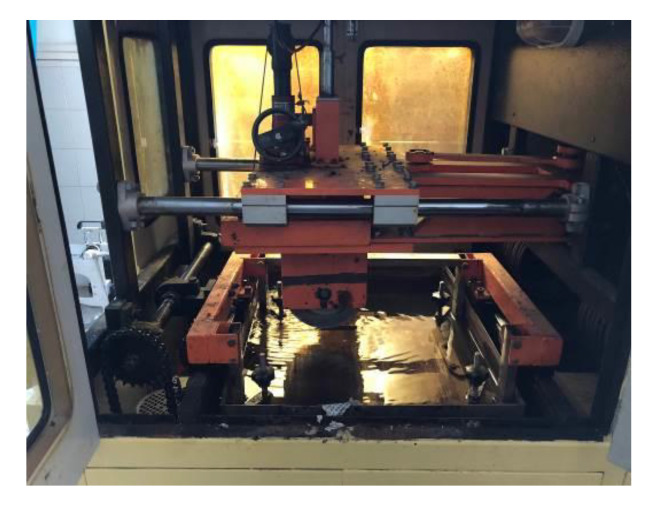
Abrasion tester.

A self-developed abrasion tester (Figure 13) was adopted, and the abrasion time was used to characterize the running time of the tire on the actual pavement. The abrasion tester is mainly composed of a kneading wheel and a horizontal plate that can carry samples. When the tester is in operation, the plate can move laterally and repeatedly, while the kneading wheel moves vertically and repeatedly. Table 9 lists the operation parameters of the abrasion tester.

Each sample was placed on a horizontal plate of the abrasion tester and fastened by a clamp. Samples, loaded at 0.7 MPa, were taken out every 1 h to measure the stress acting at the contact interface and kneaded for 12 h. In this study, to enhance the abrasion effect, the test environment was set at normal temperature under an overflowing water condition.

### 6.2. Analysis of Abrasion Test Results

Different grooving parameters lead to different attenuation laws of the concrete pavement in the process of abrasion, which can be fitted using an appropriate mathematical model, and the relevant parameters of the mathematical model can reflect this difference.

**Figure 14:**
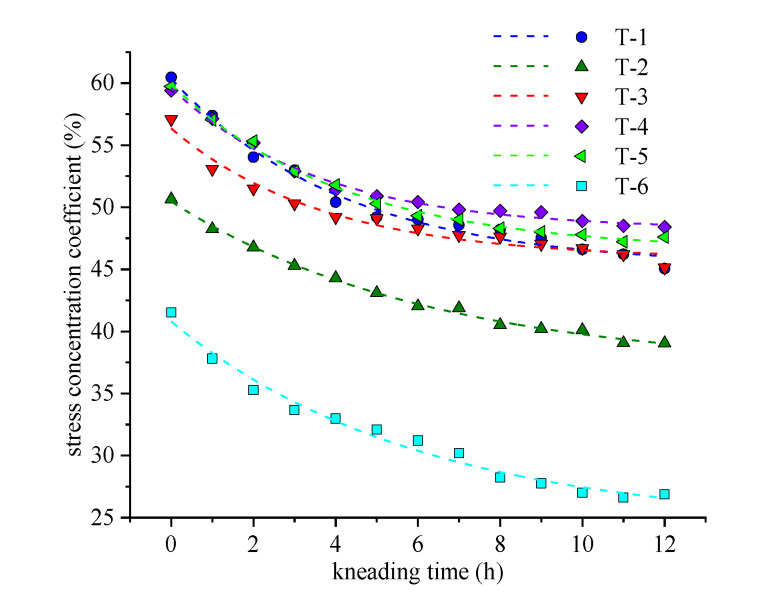
Fitting curve of stress concentration coefficient.

**Figure 15:**
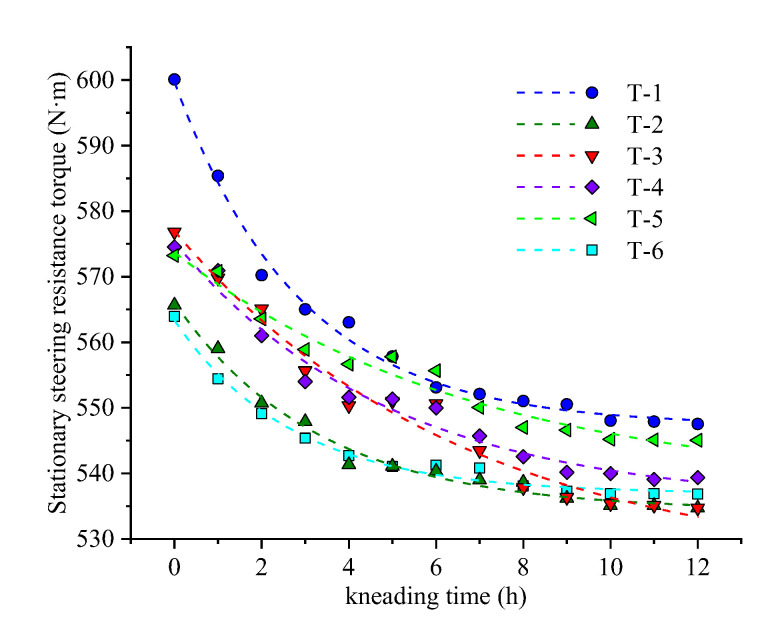
Fitting curve of stationary steering resistance torque.

According to the experimental analysis, the skid-resistance performance attenuation curve of the curved textured pavement is nonlinear and exponential. In this study, an asymptotic model was selected, as expressed in Equation (12). Figure 14 and Figure 15 show the fitting curves. Table 10 and Table 11 list the regression parameters of the fitting curve. The R^2^ are greater than 0.95 in all cases, which can better characterize the attenuation law of the evaluation indicators.

(12) $$y=Ae^{Bx}+C$$

where *y* is the value of the evaluation indicator; *x* is the abrasion time; A, B, and C are regression parameters, where A + C, C, A, B represent the initial value, final value, attenuation amplitude, and rate of reaching stable state of the indicators, respectively.

For different combinations of the texture parameters, the initial value, attenuation rate, and final value of the stress concentration factor are different. As shown in Figure 14 and Table 10, the indicator values of each sample decrease rapidly at the initial stage and then gradually tend to stabilize. Based on the value of the parameter B, the attenuation speed of T-1, T-3, T-4, and T-5 is higher than T-2 and T-6, which indicates that the greater the density of the curved groove, the more significant the stress concentration effect and the faster the abrasion of the pavement structure. The initial value order of the stress concentration coefficient is T-1 > T-5 > T-4 > T-3 > T-2 > T-6 and the values of T-1, T-5 and T-4 is similar. The initial values of curved groove interface are approximately in the range of 50–60%, while that of the rectangular grooved pavement is only 43%. The initial value of the evaluation indicator of the curved textured pavement is greater than that of the rectangular grooved pavement. Therefore, the meshing friction between the tire and the curved groove is more significant, which can prove that the texture roughness of the initial curved groove is better than that of the rectangular groove. The final values of T-1, T-3, T-4, and T-5 are significantly higher than those of T-2, indicating that the higher the groove density, the better the skid-resistance performance after abrasion. Meanwhile, the attenuation amplitude of T-3, T-4, and T-5 is less than that of T-1, which indicates that the spacing provided between the continuous curved grooves helps disperse the large stress, and the attenuation amplitude of the stress concentration coefficient can be effectively alleviated. Therefore, from the perspective of anti-skid performance, T-4 and T-5 are recommended, and the GGW is 50~70 mm.

As shown in Figure 15 and Table 11, the attenuation rate of the steering resistance torque in the early stage is higher than that in the later stage. The initial values of T-2, T-3, T-4, and T-5 are similar and greater than T-6 but significantly less than that of T-1. This proves that the groove density has a significant effect on the initial steering resistance moment. The greater the texture density, the greater the lateral force acting on the tire. Combined with the analysis of the friction mechanism between the textured pavement and the tire, the groove spacing has an interference effect at the tire–pavement contact interface, which reduces the effect of the lateral torque, yields a moderate initial index value, and reduces the driving shimmy on the curved grooved pavement. The order of final value is T-1 > T-5 > T-4 > T-3 > T-2, and T-6 is slightly higher than those of T-2, T-3, and T-4, which indicates that after abrasion, the appropriate texture parameters can help improve the driving stability of the textured pavement and maintain the steering resistance moment at a low level. Therefore, from the perspective of driving stability, T-2, T-3, and T-4 are recommended.

Considering the skid resistance performance and driving stability of the pavement, setting certain groove spacing can help effectively reduce the stationary steering resistance torque. In combination with the GGW, the skid resistance performance of the pavement can be improved. Therefore, a groove dimension scheme with a groove width of 8 mm, depth of 3 mm, spacing of 15 mm, and GGW of 50 mm is recommended.

## 7. Engineering Verification

**Figure 16:**
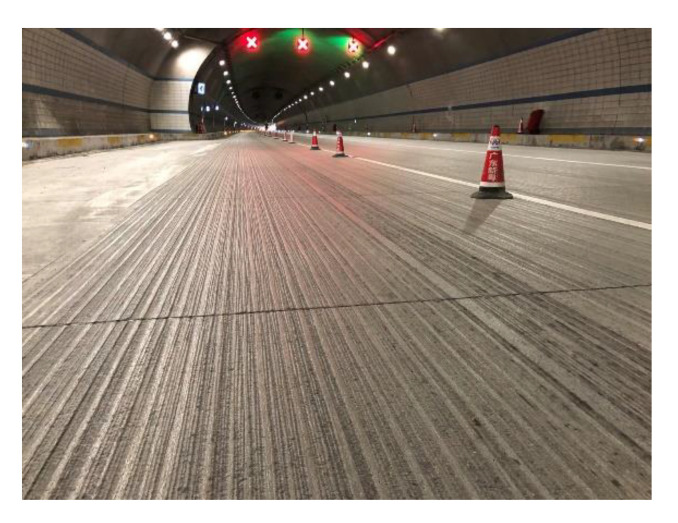
Textured pavement.

The results of this study were applied in Wang Bei Ao Tunnel (3751 m left and 3713 m right) of Jiang Luo Highway (Jiangmen to Luoding Highway) in Guangdong Province, China. This project was completed in December 2016 and opened to traffic on 26 December 2016. In this project, P.O42.5 cement (initial setting time 235 min, final setting time 287 min, 7-day bending strength 4.95 MPa, 7-day compressive strength 38.1 MPa) was used for the tunnel concrete pavement, with a dosage of 378 kg. The granite aggregates (10–30 mm, 10–20 mm, and 5–10 mm aggregates with a weight ratio of 55%, 35%, and 10%, respectively) were used. The water–cement ratio was 0.37 and the external additive, CNF-3 water reducing agent (air-entraining, slow setting, and high efficiency water reduction agent) was used with a dosage of 7.6 kg. Considering the low design speed of the tunnel pavement, a higher level of stability and comfort was required for the project, so the texture parameter T-4 (W: 8 mm, D: 3 mm, GS: 15 mm, GGW: 50 mm) was chosen for the concrete pavement. The skid-resistance performance of highway cement pavements is mainly evaluated in terms of structure depth, the sideway-force coefficient, etc., of which the sideway-force coefficient is a more commonly used evaluation method. However, the test vehicle has strict requirements on the length of the road section and driving speed. As a post-evaluation, the sideway-force coefficient cannot provide guidance in the design stage. It is difficult obtain the sideway-force coefficients for some special road sections (such as cement ramps and toll plaza pavements). In addition, to guide the design of the texture parameters in the construction process, it is necessary to evaluate the skid resistance of the concrete pavement in the process of laboratory testing. In this study, the skid resistance performance of the optimized pavement (W: 8 mm, D: 3 mm, GS: 15 mm, GGW: 50 mm), built in 2017, was tracked, as shown in Figure 16, and the correlation between the stress concentration coefficient and the transverse force coefficient was studied.

**Figure 17:**
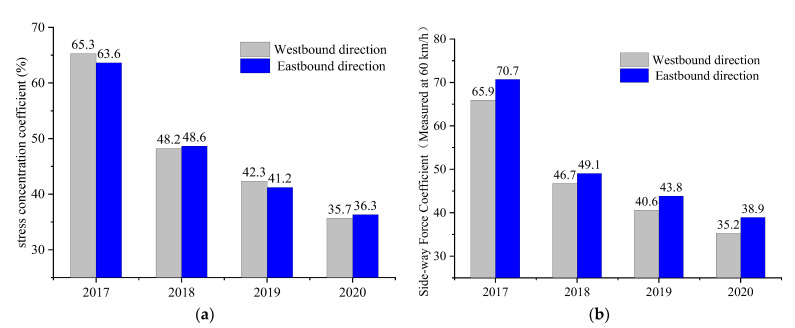
Tracking data: (**a**) stress concentration coefficient; (**b**) sideway-force coefficient (60 km/h).

A pressure film test system was used to carry out an annual inspection of highway pavements. A tire loading test was conducted at the same point every year to obtain the tire–pavement contact stress distribution. Figure 17 shows the results of the test conducted during the 2017–2020 period.

Overall, the decrease percentages of the stress concentration coefficient of westbound and eastbound direction in the first year are 57.8% and 55.0%, respectively, whereas the corresponding values of the SFC are 62.1% and 68.0%. Under the effect of significant abrasion, the skid-resistance performance decreases rapidly in the initial stage, consistent with the laboratory test results.

**Figure 18:**
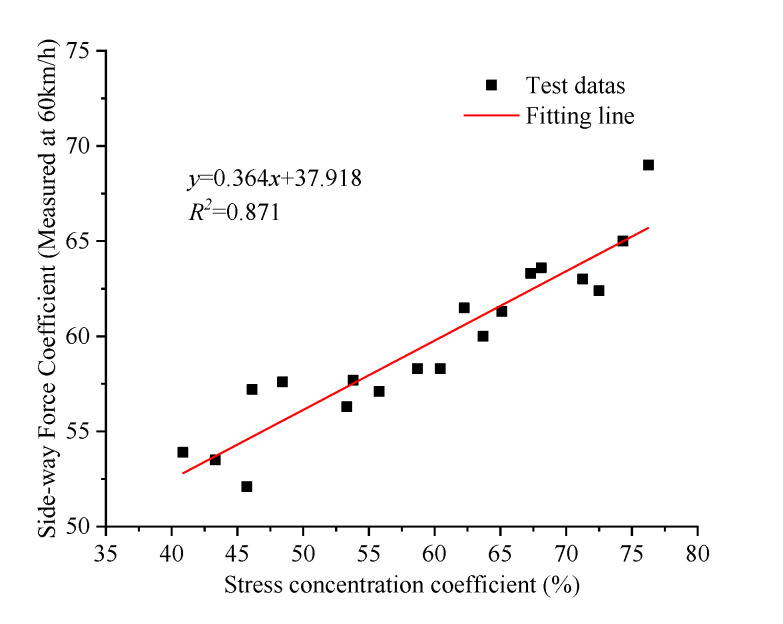
Relationship between stress concentration and SFC.

Figure 18 shows the correlation analysis results of the stress concentration factor and transverse force coefficient. The R^2^ of the test data is 0.871, indicating a good linear correlation between the tire contact stress concentration coefficient and sideway-force coefficient. Therefore, the stress concentration coefficient based on the friction mechanics can be used to characterize the skid-resistance of textured concrete pavements.

## 8. Conclusions

This study was conducted to optimize the texturing parameters of concrete pavement considering both the skid resistance and the driving stability. The work carried out by this study yields the following conclusions:

(1) The actual contact stress between the tire and the pavement can be characterized by the Weibull model. The discrete degree of the contact stress of different pavements can be ranked (from high to low) as follows: asphalt pavement (AC-16), curved grooved pavement, rectangular grooved pavement, and concrete pavements with no grooves.
(2) The compact texture structure of the textured pavement improved the friction resistance but reduced the driving stability. Theoretical and experimental analyses showed that the stationary steering resistance torque based on the measured stress could effectively help evaluate the steering effect of textured pavements on tires.
(3) The most important factors influencing the texture depth, stress concentration coefficient, and steering resistance torque were the groove depth, groove width, and groove spacing. Through the analysis of variance, we found that each texture parameter had a significant influence on the stress concentration coefficient and stationary steering resistance torque.
(4) An asymptotic attenuation model successfully described the attenuation laws of the stress concentration coefficient and stationary steering resistance torque. Based on the results of orthogonal and abrasion resistance tests, we suggest a sample with optimal dimensions (8 mm in width, 3 mm in depth, 15 mm in spacing, and 50 mm in groove group width) for balancing skid-resistance performance and driving stability.
(5) The stress concentration coefficient and SFC (measured at 60 km/h) exhibited a good linear correlation, indicating that the stress concentration coefficient can effectively characterize the skid-resistance performance of textured concrete pavements.

Based on the requirements of the practical engineering project, this study is conducted to investigate the combination of texture parameters of cement pavement with longitudinal grooves for balancing skid-resistance performance and driving stability. The further study will be focused on the effects of groove direction, tire pattern form, tire structure type, tire load, and other factors on driving stability and braking performance. Moreover, the mix design of the wear resistance of concrete pavement is also an important factor that affects the durability of the skid resistance of concrete pavement and will also be systematically investigated in future study.

## Figures and Tables

**Table 1:** Test results of stress distribution on different contact interfaces.

Contact Interface	*a*	*b*	*c*	*R* ^2^
Asphalt pavement without grooves	0.2068	0.9199	0.6558	0.999
Curved grooves	0.1987	1.4898	0.7052	0.998
Rectangular grooves	0.1645	1.2691	0.8465	0.998
Concrete pavements with no grooves	0.1809	0.4947	0.9754	0.999

**Table 2:** Cement parameters.

Initial Setting Time/min	Final Setting Time/min	Seven-Days Bending Strength/MPa	Compressive Strength/MPa
235	287	4.95	38.1

**Table 3:** Proportion of mix.

Material	Cement	Sand	10–30 mm	10–20 mm	5–10 mm	Water	CNF-13	Water–Cement Ratio
Amount/(kg)	378	691	706	449	128	140	7.6	0.37
Weight ratio	1	1.828	1.868	1.188	0.34	0.37	0.02	

**Table 4:** Factors and levels of the orthogonal test.

Test No.	W/mm	D/mm	GS/mm
1	6	1	0
2	6	2	15
3	6	3	25
4	8	1	15
5	8	2	25
6	8	3	0
7	10	1	25
8	10	2	0
9	10	3	15

**Table 5:** Orthogonal test results.

Test No.	Texture Depth/mm	Stress Concentration Coefficient/%	Stationary Steering Resistance Torque/N·m
1	0.72	47.64	531.89
2	0.80	52.63	555.31
3	0.86	55.33	607.80
4	0.76	51.84	540.62
5	0.64	51.90	640.19
6	1.02	60.79	591.57
7	0.61	47.43	547.07
8	0.72	53.09	554.13
9	1.08	59.32	549.32

**Table 6:** Range analysis of the orthogonal test results.

Results		Level Factors		
		W	D	GS
Texture depth	*K*_11_	2.38	2.09	2.46
	*K*_21_	2.42	2.16	2.64
	*K*_31_	2.41	2.96	2.11
	*Rj*	0.01	0.29	0.18
Stress concentration coefficient	*K*_12_	155.6	146.91	161.52
	*K*_22_	164.53	157.62	163.79
	*K*_32_	159.84	175.44	154.66
	*Rj*	2.98	9.51	3.04
Stationary steering resistance torque	*K*_13_	1695	1619.58	1677.59
	*K*_23_	1772.38	1749.63	1645.25
	*K*_33_	1650.52	1748.69	1795.06
	*Rj*	40.62	43.35	49.94

**Table 7:** Analysis of variance table.

Evaluation Index	Factors	Sum of Square between Groups	Degree of Freedom	*f* Value	Significance Degree
Texture depth	W	2.89 × 10^−4^	2	0.14	not significant
	D	1.56 × 10^−1^	2	77.02	moderately significant
	GS	4.84 × 10^−2^	2	23.95	moderately significant
Stress concentration coefficient	W	13.30	2	19.10	moderately significant
	D	138.47	2	198.82	highly significant
	GS	15.06	2	21.63	moderately significant
Stationary steering resistance torque	W	2535.11	2	19.29	moderately significant
	D	3731.48	2	28.39	moderately significant
	GS	4143.12	2	31.53	moderately significant

**Table 8:** Test specimen parameters.

No.	W	D	GS	GGW
T-1	8	3	0	8
T-2	8	3	15	8
T-3	8	3	15	30
T-4	8	3	15	50
T-5	8	3	15	70
T-6	Parameters of rectangular groove: 8 mm in width; 3 mm in depth; 15 mm in spacing.			

**Table 9:** Operating parameters of abrasion tester.

Lateral Speed (cm/min)	Wheel Movement Frequency (times/min)	Pressure (MPa)
10	42 ± 1	0.7

**Table 10:** Attenuation parameter of stress concentration coefficient.

Parameter Combination	A	B	C	R^2^
T-1	15.166	−0.238	45.184	0.983
T-2	13.428	−0.159	37.039	0.995
T-3	10.511	−0.270	45.835	0.965
T-4	11.421	−0.279	48.196	0.991
T-5	13.446	−0.240	46.467	0.995
T-6	16.420	−0.167	24.369	0.980

**Table 11:** Attenuation parameter of stationary steering resistance torque.

Parameter Combination	A	B	C	R^2^
T-1	52.416	−0.347	547.305	0.992
T-2	31.743	−0.304	534.328	0.988
T-3	51.984	−0.152	525.004	0.977
T-4	40.137	−0.203	535.196	0.978
T-5	36.042	−0.146	537.679	0.970
T-6	26.407	−0.371	536.948	0.990

## Data Availability

The data presented in this study are available on request from the corresponding author.
